# Editorial: Occupational Medicine: Disease Risk Factors and Health Promotion

**DOI:** 10.3389/fpubh.2021.819545

**Published:** 2022-01-25

**Authors:** Vladimir Jurisic, Caterina Ledda, Nicola Mucci, Silvio Tafuri, Luigi Vimercati

**Affiliations:** ^1^Faculty of Medical Sciences, University of Kragujevac, Kragujevac, Serbia; ^2^Occupational Medicine, Department of Clinical and Experimental Medicine, University of Catania, Catania, Italy; ^3^Department of Experimental and Clinical Medicine, University of Florence, Florence, Italy; ^4^Department of Biomedical Science and Human Oncology, Aldo Moro University of Bari, Bari, Italy; ^5^Interdisciplinary Department of Medicine, University of Bari, Bari, Italy

**Keywords:** COVID-19, prevention, worker, burden, cancer, injury, exposure, workplace

Occupational Medicine has always been concerned with preventing health problems caused by working conditions, and with promoting and maintaining the highest level of physical, mental, and social wellbeing of workers in all occupations (1–3). In the last few decades, with an aging work population and arise in sickness and absenteeism with the associated financial impacts on organizations, workplace health promotion has become a top priority. Regarding this issue, some research groups have carried out studies on health promotion. In a survey, Di Lorenzo et al. compared healthcare workers with other employees on adherence to the Mediterranean Diet and blood lipid profile; their results showed the preventive contribution in the context of periodic health surveillance by occupational physicians. Research group of Garcia-Rojas carried out a non-randomized company-based trial to evaluate a worksite health promotion program in seven Mexican companies. The investigation confirmed that a promotion activity carried out in an occupational medicine context could be helpful to reduce high blood pressure, in particular, among diabetic workers (Garcia-Rojas et al.). Hanson et al. pointed out that it is appropriate to intervene on some groups of workers such as those working in construction and homecare, who seem to have higher modifiable and non-modifiable risk factors compared to the general population. Hermann et al. and Merati et al. reported that it is appropriate to carry out strategies based on occupational medicine to mitigate the risks that may interfere with the overall health of workers. In Italy, a good practice implemented by Tuscany Region on anti-meningococcal vaccination was highlighted among the healthcare workers (Gattini et al.).

The coronavirus disease 2019 (COVID-19) pandemic has also changed the public opinion on the need for safe practices in the workplace and has focused the attention of governments and institutions worldwide on the fundamental role that the occupational safety and health services play. In particular, healthcare workers have been affected by the various waves of severe acute respiratory syndrome coronavirus 2 (SARS-CoV-2) even with psychological harm (Al-Kuwari et al.; Costantino et al.; Dudine et al.); this has also affected other workers (Haan et al.). The COVID-19 has also impacted the transport and the environment (De Maria et al.).

Naithani et al. found a low baseline knowledge of occupational health hazards and demonstrated that an appropriate form of training can reduce injuries, resulting from occupational hazards and ensure a healthy workforce that can contribute toward a positive impact on national economies.

Moreover, more attention should be given to the long-term effects of ionizing radiation. The occupational protection of radiographic inspection workers should be strengthened to reduce and/or avoid occupational injuries, thus, protecting the health and safety of workers (Hao et al.). Furthermore, symptoms of low-frequency time-varying magnetic fields were studied by Bravo et al.. The investigation did not show any association between the occurrence of reversible subjective symptoms, including the more specific “core symptoms.” On the other hand, the role played by occupational stress appears to be not negligible (Bravo et al.). Psychosocial issues were also addressed. Alabi et al., in a wide-ranging review, analyzed the interventions to improve job satisfaction and job retention among oncologists, and, therefore, to improve patient care. Healthcare professionals are increasingly affected by events that can affect mental health, and, therefore, it is advisable to evaluate various hospital profiles and settings. In fact, Primary Care nurses showed higher levels of work engagement and a lower perception of psychosocial risks than Emergency nurses (García-Iglesias et al.). This finding was also confirmed by Rostami et al. in a cross-sectional study carried out on nurses, midwives, and administrative workers.

Not only are healthcare workers affected by psychosocial disorders, various studies also reported burnout among Private Security Staff (Veljković et al.) and a reduction of psychosocial factors in optimal work conditions among workers subjected to heavy physical work (Sala et al.).

Another issue is balancing work and family demands. Family and job responsibilities may affect many aspects of health, and sleep is an important issue. The reduction of work-family conflict improves sleep quality (Silva-Costa et al.).

In occupational medicine, an emerging problem is the underestimated lack of work that can cause damage to the health of workers. Costa et al. highlighted that females are associated with higher stress levels, pointing out the relevance of specific interventions in the context of health promotion programs, especially in order to mitigate stress in more susceptible subjects.

Finally, workplace interventions on the health, safety, and lifestyle of workers can be a model to export to developing populations. In the last few years, new exposure assessment methods in occupational settings have led to significant improvements in the quality of scientific studies and other measures to promote and support workers' health. On the other hand, several persistent as well as emerging issues (i.e., the increase in chronic disease and mental ill health; work-related stress, technological advances, and new ways of working such as smart working) justify the continuing need for novel strategies to better assess the exposure to old and new occupational risk factors.

## Author Contributions

All authors listed have made a substantial, direct, and intellectual contribution to the work and approved it for publication.

## Conflict of Interest

The authors declare that the research was conducted in the absence of any commercial or financial relationships that could be construed as a potential conflict of interest.

## Publisher's Note

All claims expressed in this article are solely those of the authors and do not necessarily represent those of their affiliated organizations, or those of the publisher, the editors and the reviewers. Any product that may be evaluated in this article, or claim that may be made by its manufacturer, is not guaranteed or endorsed by the publisher.
